# Dengue among Suspected Patients Admitted to Department of Medicine of a Tertiary Care Centre: A Descriptive Cross-sectional Study

**DOI:** 10.31729/jnma.8033

**Published:** 2023-02-28

**Authors:** Dinesh Kumar Lamsal, Prem Shankar Chaurasiya, Akash Khatri, Swekchha Karki, Soniya Singh, Gomik Shilpakar, Raj Kumar Sangroula

**Affiliations:** 1 Department of Family Medicine, Civil Service Hospital, New Baneshwor, Kathmandu, Nepal; 2 Department of Internal Medicine, Kalaiya Provincial Hospital, Kalaiya, Bara, Nepal; 3 Department of Internal Medicine, Civil Service Hospital, New Baneshwor, Kathmandu, Nepal; 4 Department of Laboratory, Civil Service Hospital, New Baneshwor, Kathmandu, Nepal; 5 Department of Public Health, Little Buddha College of Health Science, New Baneshwor, Kathmandu, Nepal

**Keywords:** *dengue virus*, *public health*, *tertiary care centre*

## Abstract

**Introduction:**

Dengue virus incidence has been increasing trends in every year due to the expansion of the vectors *Aedes aegypti* and *Aedes albopictus.* The objective of this study was to find out the prevalence of dengue among suspected patients admitted to the department of medicine of a tertiary care centre.

**Methods:**

A descriptive cross-sectional study was conducted among patients admitted to the medicine department from 30 September 2022 to 30 December 2022 after obtaining ethical approval from the Institutional Review Committee (Reference number: 019/2022). Demographic, clinical characteristics and laboratory profiles were collected from dengue patients by using a structured questionnaire. Convenience sampling method was used. Point estimate and 95% Confidence Interval were calculated.

**Results:**

Among 500 patients, 242 (48.40%) (40.66-56.14, 95% Confidence Interval) were found to be dengue positive. The average age of the enrolled patients was 39.13±20.64 years. Most dengue fever patients were diagnosed in the category of dengue with a warning sign of 234 (96.69%). The mean hospital stay of dengue patients was 4.05±2.03 days, 229 (94.62%) of patients stayed less than 7 days before discharge.

**Conclusions:**

The prevalence of dengue among suspected patients admitted to the department of medicine is found to be higher than in other similar studies done in similar settings. Patients with clinical symptoms and laboratory findings corroborating with dengue should undergo early diagnosis and facilitate prompt treatment in individual patients.

## INTRODUCTION

Dengue virus (DENV) infection is an arthropod-borne virus that is transmitted to humans by the bite of infected female mosquito species mainly *Aedes aegypti* and *Aedes albopiticus,* with local variations in risk influenced by climate parameters like social and environmental factors.^1^ There are four distinct serotypes of the virus that cause dengue (DENV-1, DENV-2, DENV-3 and DENV-4) with major responsibility for the epidemic occurring at 2-3 years intervals as epidemiological trends.^2^

Dengue incidence has increased in recent years due to the expansion of vectors *Aedes aegypti* and *Aedes albopictus,* as well as the movement of people and the introduction of imported cases.^3^ Nepal faced an unprecedented dengue outbreak in 2022 with more than 53,951 cases and 62 deaths.^4^ Community based study found substantial expansion in Western and Far Western Terai region of Nepal but there are limted study done in hospital.^5^

The objective of this study was to find out the prevalence of dengue among suspected patients admitted to the department of medicine of a tertiary care centre.

## METHODS

This was a descriptive cross-sectional study conducted in Civil Service hospital, Kathmandu, Nepal from 30 September 2022 to 30 December 2022 after ethical approval from the Institutional Review Committee (Reference number: 019/2022). The study population were all the suspected patients, who presented to the HDU, ICU and medical ward within study period were included. Those patients with missing biochemical and serological data wre excluded. Convenience sampling was used. The sample size was calculated by using the following formula:


$$\begin{array}{ll} \text{n} & ={\text{Z}}^{2}\times \frac{\text{p}\times \text{q}}{{\text{e}}^{\text{2}}}\\ & =1.96^{2}\times \frac{0.23\times 0.77}{0.04^{2}}\\ & =426 \end{array}$$

Where,

n = minimum required sample size
Z = 1.96 at 95% of Confidence Interval (CI)
p = prevalence taken from previous studies as 23.09%^6^
q = 1-p
e = margin of error, 4%

The calculated minimum sample size was 427. However, 500 cases were included in the study.

In this study, dengue is suspected when a high fever (40°C/104°F) is accompanied by 2 of the following symptoms during the febrile phase (2-7 days): severe headache, pain behind the eyes, muscle and joint pains, nausea, vomiting, swollen glands, rash.^1^ For confirmation, viruses were detected by testing for a protein made by the virus called NS1. The presence of IgM indicates recent DENV infection. IgG antibody levels were also taken for confirmation.^1^

Cases were confirmed by clinical outcomes and dengue serology tests. After taking informed consent, daily history taking through patient or patient party followed by physical examination at the bedside were done. The necessary laboratory tests were sent to the laboratory. The demographic variables (Age, sex, address) were carefully noted. All cases presenting as well as new symptoms and signs were recorded in a structured questionnaire. Different subtypes of Dengue infections (dengue with no warning sign, dengue with a warning sign, and severe dengue) were categorized according to WHO criteria.^13^ Laboratory data about the serological status of Dengue (NS1, IgG, IgM) and platelets, leukocyte count, hematocrit and liver biochemistry (SGPT, SGOT) level were carefully noted in the questionnaire.

Data were entered in Microsoft Excel 2013 and analysed using IBM SPSS Statistics version 22.0. Point estimate and 95% CI were calculated.

## RESULTS

Among 500 suspected dengue patients, 242 (48.40%) (40.66-56.14, 95% CI) dengue patients were confirmed by serology tests and clinical outcomes in our study. The mean age of participants was 39.13±20.64 years and 58 (23.96%) of patients belonged to the age group 20-29. The positive female and male ratio were 1:2 (Table 1).

**Table 1:** Demographic profile confirmed seropositive cases of dengue (n= 242).

Characteristics	n (%)
**Sex**	
Male	128 (52.89)
Female	114 (47.11)
**Age (years)**	
<10	7 (2.89)
10-19	26 (10.74)
20-29	58 (23.97)
30-39	43 (17.77)
40-49	48 (19.83)
50-59	18 (7.44)
60-69	10 (4.13)
70-79	17 (7.02)
80-89	12 (4.96)
90-99	3 (1.24)

Most of the dengue fever patients had got a diagnosiscategory of dengue with warning signs 234 (96.69%)followed by severe dengue wasencountered to 8(3.31%) of all admitted cases (Table 2).

**Table 2:** Dengue fever types, hospital stay and dengue serology (n= 242).

Characteristics	n (%)
**Type**	
Dengue with warning signs	234 (96.69)
Severe dengue	8 (3.31)
**Hospital stays (days)**	
≤7	229 (94.63)
>7	13 (5.37)
**Serology**	
NS1 antigen positive	235 (97.11)
IgM positive	26 (10.74)
IgG	15 (6.20)

Furthermore, mean and standard deviation of total WBC count, platelets, HCT, SGPT and SGOT of dengue patients on Day 1 and after Day 7 of hospitalization were also calculated (Table 3).

**Table 3:** Laboratory characteristics of patients with dengue at day 1 and day 7 (n= 242).

Parameters	Day 1 (Mean±SD)	Day 7 (Mean±SD)
Total WBC count	4848.02 ±4286.52	5401.08±2774.37
Platelets	105330.87±75774.57	148005.15±114303.82
HCT	40.09±6.63	41.46±25.05
SGPT	145.62±186.63	165.27±148.49
SGOT	284.60±446.72	190.94±171.51

Among the total seropositive cases, fever was seen as the major clinical symptoms in 216 (89.26%) of the patients followed by myalgia 149 (61.57%) with common complaints during an initial presentation at the hospital (Table 4).

**Table 4:** Signs and symptoms of seropositive cases of dengue (n= 242).

Symptoms/signs	n (%)
Fever	216 (89.26)
Myalgia	149 (61.57)
Headache	148 (61.16)
Lethargy	138 (57.02)
Nausea and vomiting	120 (49.59)
Periorbital pain	99 (40.91)
Decreased appetite	61 (25.21)
Bleeding	57 (23.55)
Abdominal pain	54 (22.31)
Loose stool	42 (17.35)
Rash	31 (12.81)
Hypotension	27 (11.16)
Cough	8 (3.30)
Ascites	8 (3.31)
Hepatosplenomegaly	8 (3.31)
CNS manifestations	7 (2.89)
Pleural effusion	7 (2.89)
Decreased urine output	4 (1.65)
Itching	4 (1.65)

The liver enzyme SGPT was raised (>50 IU/L) in 139 (57.44%) patients while SGOT was found raised (>45 IU/L) in 195 (80.58%) of dengue fever cases. Moreover, leucopenia was found in 121 (50.00%) of the total dengue patients (Table 5).

**Table 5:** Clinical characteristics of dengue patients (n= 242).

Clinical characteristics	n (%)
**Platelets count**	
Mild thrombocytopenia (101000 to 51000 /cumm	72 (29.75)
Moderate thrombocytopenia (51000 to 21000) /cumm	45 (18.60)
Severe thrombocytopenia < 21000/cumm	14 (5.79)
Normal (>101000/cumm)	111 (45.87)
**Leucocyte count status**	
Leucopenia (<4000/cumm)	121 (50.00)
Normal	121 (50.00)
**Hematocrit level**	
Raised (≥50)	26 (10.74)
Normal (<50)	216 (89.26)

## DISCUSSION

This study showed that high prevalence of DENV was found to be 242 (48.40%) at tertiary care hospital as compared to the other reported hospital setting studies in Nepal. A dengue study conducted in the western Terai region of Nepal in 2012 reported that the increased prevalence of DENV in tertiary care hospitals might be due to the expansion of the vectors *Aedes albopictus,* with local variations in risk influenced by climate parameters i.e., social and environmental factors as well as the movement of people in highly endemic areas of DENV to Kathmandu valley.^5^ This study showed high positivity rate than the study conducted in Thailand, 2011(9.80 %)which might be due to the difference in the geographical distribution in Nepal.^7^ However, the present study result was consistent with some of the previous findings from Nepal conducted by EDCD, 2022.^8^

Our present study showed that the common age group infected by dengue infection was 20-29 yearsfollowed by 40-49 age groups with corresponding proportions of 23.97%, 19.83%, and 17.77% respectively. This is in accordance with similar findings from the western part of Uttar Pradesh.^9^ However, the pediatric population, age groups 7-12 and 13-18 years were found more susceptible to dengue fever (DF).^10^ A study conducted in Lahore teaching hospital found that (81.10%) of dengue patients belong to males.^11^

Our study showed that (96.69%) of patients had dengue with a warning sign and only (3.31%) of patients were suffering from severe dengue based on WHO classification. The present study findings are very similar to a study done in Bangladesh, 2022 where (94.10%) had non-severe dengue and the remaining (5.90%) had severe dengue.^12^ On the contrary, a Nepalese study conducted in a similar setting in 2022 revealed that dengue with warning signs was seen in (27.30%)^13^ which is very low as compared to the current study. The mean hospital stay was 3.07±1.78 in a study conducted at Dhaka hospital.^12^ The current study shows a slightly more prolonged stay of 4.05 days than before the relevant study.

Among the dengue cases, the most common clinical features were fever, myalgia, headache, lethargy, nausea and vomiting, and periorbital pain during the outbreak of dengue in Nepal.^14^ On the contrary, less common symptoms reported by the current study include decreased appetite, bleeding, abdominal pain and loose stool. However, the severities of these clinical symptoms are diverse in each individual patient depending on their immune system such as disease associated with different serotypes or with more than one serotype.^15^

In the present study, dengue patients had thrombocytopenia and elevated liver enzymes level which is similar to the study conducted in Northwest Ethiopia.^16^ A similar finding from a study conducted previously in Nepal recorded that platelets <100,000 level were significantly linked with causing dengue infection which is consistence with the present study.^17,18^ Therefore, previous study findings recommended that thrombocytopenia and raised transaminases are good markers for the assessment of the severity of dengue viral infection.^19^

Similarly, haematological and biochemical parameters i.e., total WBC count, Platelets and SGPT of dengue patients on Day 1 and after Day 7 of hospitalization increased among the admitted patients compared to previous findings in tertiary care hospital in Dhaka city.^12^ Furthermore, previous literature showed that alteration of haematological, parameters merge with others i.e., clinical and laboratory markers could help physicians to early diagnosis of dengue on the first day of admission as well as prevent further development of severe dengue hemorrhagic fever.^20^

Since this study was conducted in a single centre using convenience sampling so the results might not be generalizable to a larger population.

## CONCLUSIONS

The prevalence of dengue among patients admitted to the medicine department is found to be higher than in other similar studies done in similar settings. The concerned authorities should strengthen province, local and central wise government by surveillance network i.e. serological, molecular and integrated vector control programs for prevention and control of DENV in the coming year.
